# Supplementary material for: Widespread Distribution of Luteinizing Hormone/Choriogonadotropin Receptor in Human Juvenile Angiofibroma: Implications for a Sex-Specific Nasal Tumor
Source: Cells. 2024 Jul 19;13(14):1217. doi: 10.3390/cells13141217 (PMC11274802; doi:10.3390/cells13141217)
Supplement: Supplementary file 1 [file cells-13-01217-s001.zip › cells-3063262-supplementary.pdf]

Supplementary Materials for

Widespread Distribution of Luteinizing Hormone/Choriogonadotropin Receptor in Human Juvenile Angiofibroma: Implications for a Sex-Specific Nasal Tumor

Silke Wemmert [1†], Martina Pyrski [2†], Lukas Pillong [1], Maximilian Linxweiler [1], Frank Zufall [2], Trese Leinders-Zufall [2*], and Bernhard Schick [1*]

[1] Department of Otorhinolaryngology, Head and Neck Surgery; Saarland University Medical Center, 66424 Homburg, Germany; silke.wemmert@uks.eu (S.W.); lukas.pillong@uks.eu (L.P.); maximilian.linxweiler@uks.eu (M.L.); b.schick@mx.uni-saarland.de (B.S.)

[2] Center for Integrative Physiology and Molecular Medicine (CIPMM), Saarland University, 66424 Homburg, Germany; martina.pyrski@uks.eu (M.P.), frank.zufall@uks.eu (F.Z.); trese.leinders@uks.eu (T.L.-Z.)

This PDF file includes: Figs. S1

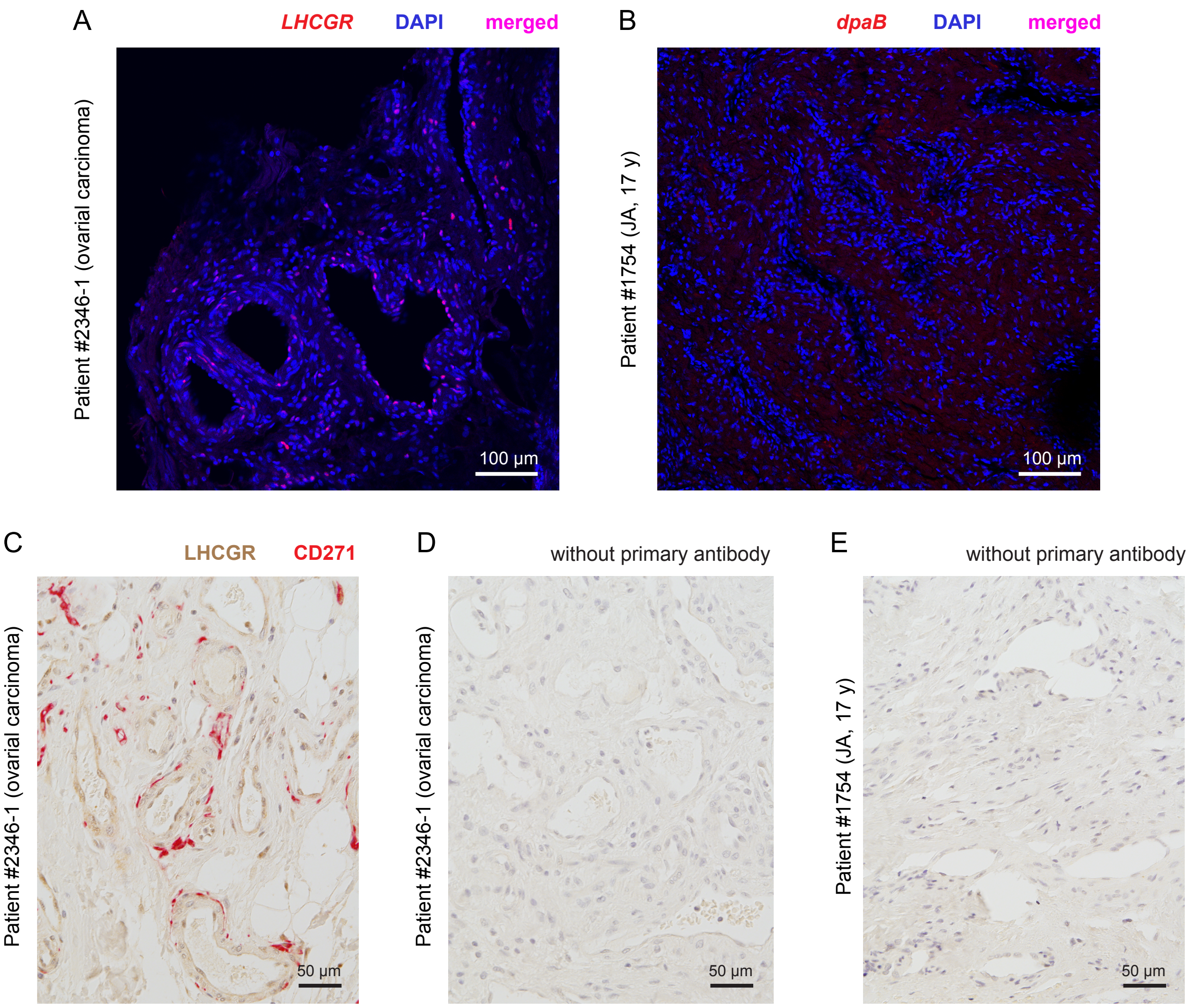

Figure S1. Validation of RNAscope and immunohistochemistry experiments. (**A**) RNAscope positive control for *LHCGR* on an ovarian tissue section from an ovarian carcinoma patient (#2346-1). *LHCGR*$^{+}$ cells (*red*) are visible. DAPI (*blue*) indicates the location of nuclei. (**B**) RNAscope negative control reaction using a tissue section from JA patient #1754. The RNAscope negative control was a channel 1 probe for the *dapB* gene of Bacillus subtilis strain SMY provided by the manufacturer (Cat#320871, ACD Bio-Techne). No specific staining was detected. DAPI indicates the location of nuclei. RNAscope controls of the examples depicted in A and B were performed on the same day and with the same solutions except for the used probe. *LHCGR* RNAscope labeling from the same patient is depicted in Figure 2J - L. (**C**) As a positive control for the immunohistochemical experiments, an ovarian tissue section from the same patient (#2346-1) as in A was used. LHCGR^{+} (*brown*), CD271^{+} (*red*). (**D**) Negative control from the same tissue as in C. Both LHCGR and CD271 primary antibodies were omitted resulting in no signal for these probes. The tissue sections of C and D were performed on the same day and with the same solutions except for the presence of the primary antibodies. (**E**) Negative control in a tissue section from JA patient #1754, in which both LHCGR and CD271 primary antibodies were omitted resulting in no signal. A positive control from the same patient using anti-LHCGR is depicted in Figure 4G. (C - E) Nuclei are stained using hematoxylin.
